# Human Metapneumovirus-Induced Host microRNA Expression Impairs the Interferon Response in Macrophages and Epithelial Cells

**DOI:** 10.3390/v15112272

**Published:** 2023-11-18

**Authors:** Iván Martínez-Espinoza, Anang D. Bungwon, Antonieta Guerrero-Plata

**Affiliations:** Department of Pathobiological Sciences, School of Veterinary Medicine, Louisiana State University, Baton Rouge, LA 70803, USA; imart27@lsu.edu (I.M.-E.); abungw1@lsu.edu (A.D.B.)

**Keywords:** miRNAs, miRNA-4634, HMPV, IFN, macrophages, epithelial cells, human metapneumovirus, lung, respiratory

## Abstract

Human metapneumovirus (HMPV) is a nonsegmented, single-stranded negative RNA virus and a member of the *Pneumoviridae* family. During HMPV infection, macrophages play a critical role in defending the respiratory epithelium by secreting large amounts of type I interferon (IFN). MicroRNAs (miRNAs) are small, noncoding, single-stranded RNAs that play an essential role in regulating gene expression during normal cellular homeostasis and disease by binding to specific mRNAs, thereby regulating at the transcriptional and post-transcriptional levels with a direct impact on the immune response and other cellular processes. However, the role of miRNAs in macrophages and respiratory viral infections remains largely unknown. Here, we characterized the susceptibility of THP-1-derived macrophages to HMPV infection and the effect of hsa-miR-4634 on these cells. Transfection of an miRNA mimic and inhibitor demonstrated that hsa-miR-4634 regulates the IFN response in HMPV-infected macrophages, suggesting that HMPV induces the expression of the miRNA as a subversion mechanism of the antiviral response. This effect was not limited to macrophages, as a similar effect was also observed in epithelial cells. Overall, our results demonstrate that hsa-miR-4634 is an important factor in regulating the IFN response in macrophages and epithelial cells during HMPV infection.

## 1. Introduction

Human metapneumovirus (HMPV) is a nonsegmented, single-stranded negative RNA virus member of the *Pneumoviridae* family [1,2]. Epidemiological evidence positions HMPV as an important cause of acute respiratory tract infections in both the upper and lower respiratory tract in children, the elderly, and immunocompromised individuals [3,4]. In pediatric populations, HMPV has a global prevalence of up to 86% in children under 5 years [5,6]. Approximately one million outpatient clinic visits and 263,000 emergency visits are associated with HMPV infection every year [7]. Currently, no specific antiviral treatment or efficient vaccine is commercially available for HMPV.

During HMPV infection, alveolar macrophages and epithelial cells are fundamental factors that activate the host’s immune response in the lung [8]. Both cell types respond to the viral infection by inducing the expression of inflammatory cytokines and interferons that contribute to the recruitment of inflammatory cells and the antiviral state, shaping innate and adaptive immunity [1,9,10,11,12]. Epithelial cells are the main target population for HMPV replication [13] and are capable of producing a myriad of cytokines, including type I and type III interferons (IFNs) [14,15]. Alveolar macrophages play a crucial role in the defense against respiratory viral infections [16,17,18]. They are strategically situated on the luminal surface of the alveolar space and act as the first lung defense against respiratory pathogens. Therefore, these cells play a central role in innate host defense mainly by producing IFNs, which is critical for antiviral defense by triggering the expression of interferon-stimulated genes (ISGs) that restrict viral replication and activate other functions [19].

Furthermore, macrophages respond quickly to viral infection by producing different inflammatory cytokines [1,11,12]. In HMPV infection, macrophages contribute to IFN production in the respiratory tract, as demonstrated in an experimental mouse model [20]. However, the HMPV–macrophage interaction remains largely unknown.

MicroRNAs (miRNAs) are highly conserved small noncoding RNAs (sncRNAs), usually of 18–25 nucleotides in length, that interact with the 3′-unstranslated region (3′-UTR) of target mRNAs. They are implicated in regulating gene expression by repressing the translation of target mRNAs or promoting their degradation during cellular normal homeostasis as well as disease states [21,22]. We have previously reported the miRNA profile induced by HMPV in human monocyte-derived dendritic cells [23,24]. However, the functional effect of miRNAs induced in HMPV-infected cells remains understudied.

miRNAs are known to regulate the functions of a variety of cells [20,24]. Here, we analyzed the susceptibility of THP1-derived macrophages to HMPV infection and investigated the induction of miRNA expression. We found that hsa-miR-4634 is one of the predominant miRNAs induced in macrophages during HMPV infection. Additional functional analysis revealed that hsa-miR-4634 interferes with the interferon response in macrophages and epithelial cells. These findings represent a novel strategy by which HMPV evades the host immunity and might open new avenues for potential therapy targets in HMPV infection.

## 2. Materials and Methods

### 2.1. Cell Culture

All cell lines were obtained from the American Type Culture Collection (ATCC). The human monocytic THP-1 cell line (ATCC, TIB-202, Manassas, VA, USA) was cultured in RPMI 1640 (HyClone, Logan, UT, USA) supplemented with 10% fetal bovine serum (FBS) (Gibco, Gaithersburg, MD, USA), 2% sodium pyruvate (ThermoFisher Scientific, Waltham, MA, USA), and 1% penicillin-streptomycin (Gibco, Gaithersburg, MD, USA), herein referred as complete medium. Cells were maintained in incubation with 5% CO_2_ at 37 °C at a density of 1 × 10^6^ cells/mL and were used for experiments between passages 5 and 12. THP-1 monocytes were differentiated into macrophages by treating them with 100 ng/mL of phorbol 12-myristate 13-acetate (PMA) (Millipore Sigma, Burlington, MA, USA) for two days. The medium was replaced with fresh medium, and cells were incubated for two additional days before experiments.

LLC-MK2 cells (ATCC, CCL-7, Manassas, VA, USA), an epithelial cell line derived from the kidney of an adult monkey, were propagated in MEM/EBSS medium (Cytiva, Marlborough, MA, USA) enriched with 10% FBS (Gibco, Gaithersburg, MD, USA) and 1% penicillin-streptomycin (Gibco, Gaithersburg, MD, USA).

A549 cells, a human alveolar basal epithelial cell line derived from lung adenocarcinoma with type II alveolar epithelial cells (ATCC CCL-185C, Manassas, VA, USA), were cultivated in F12K medium (Gibco, Gaithersburg, MD, USA) supplemented with 10% FBS (Gibco, Gaithersburg, MD, USA) and 1% penicillin-streptomycin (Gibco, Gaithersburg, MD, USA), herein referred as the complete medium.

### 2.2. Virus Stocks

HMPV strain CAN97-83 was obtained from the Centers for Disease Control, Atlanta, GA, USA. The virus was propagated in LLC-MK2 cells in MEM containing 1 μg trypsin/mL (Worthington Biochemicals, Lakewood, NJ, USA) and purified by polyethylene glycol precipitation, followed by centrifugation on a 60% sucrose cushion [25,26]. The viral titer (PFU/mL) for HMPV was determined by a combined method of methylcellulose plaque assay and cell-based immunoassay in LLC-MK2 cells, as previously reported [27].

### 2.3. Viral Infection

For viral infection of macrophages, 5 × 10^5^ THP-1 cells were seeded in a 24-well plate. For epithelial cells, 2.5 × 10^5^ cells were seeded in 24-well plates. The following day, cells were infected with HMPV at different multiplicities of infection (MOI) in the presence of 1 μg/mL of trypsin (Worthington Biochemical, Lakewood, NJ, USA) in plain RPMI 1640 medium. After 2 h of viral adsorption, the inoculum was removed, and 1 mL/well of complete RPMI medium was added. At 24 h postinfection, cells or cell lysates were collected for further analysis.

### 2.4. Flow Cytometry

The expression of CD11b, HMPV, 7-Aminoactinomycin D (7-AAD), and HMPV was evaluated by flow cytometry. To quantify CD11b expression in macrophages, Fc receptors were blocked with FcR blocking reagent (Miltenyi Biotec, Gaithersburg, MD, USA), followed by the staining of anti-human CD11b phycoerythrin-conjugated antibody (eBioscience, San Diego, CA, USA) or isotype control (mouse IgG1κ, eBioscience, San Diego, CA, USA); cells were fixed with formaldehyde 1% before analysis. Cell death was determined by staining; the cells were stained for 30 min on ice with the live/dead dye 7-AAD (eBioscience, San Diego, CA, USA) and analyzed immediately after incubation. HMPV infection was assessed in the cells by surface and intracellular staining. Macrophages were detached and washed with PBS containing 0.5% BSA (PBS/BSA). For labeling, cells were incubated for 30 min at 4 °C with anti-FcR blocking (Miltenyi) to prevent nonspecific binding. Cells were washed with PBS/BSA and stained with anti-human CD11b antibody PE-conjugated (Cytek, Biosciences, Fremont, CA, USA) and mouse anti-HMPV phosphoprotein antibody (Bio-Rad, Hercules, CA, USA) for 30 min at 4 °C followed by an Alexa Fluor-488 goat anti-mouse antibody (Life Technologies Carlsbad, CA, USA, USA). To detect intracellular HMPV antigen, stained cells were fixed with Cytofix/cytoperm (BD Biosciences, Franklin Lakes, NJ, USA) and permeabilized with Perm/wash buffer (Biosciences, Franklin Lakes, NJ, USA). Permeabilized cells were subsequently stained with an anti-HMPV antibody, as mentioned above. Samples were acquired on a FACScan flow cytometer (BD Biosciences, Franklin Lakes, NJ, USA). Data analysis was performed with FlowJo v10.8 software (BD Biosciences, Franklin Lakes, NJ, USA).

### 2.5. Nucleofection

Transfection of macrophages and epithelial cells was performed by electroporation using the Nucleofector II (Amaxa, Cologne, Germany). An amount of 5 × 10^5^ cells was used for each transfection with either miRNA mimic or inhibitor (ThermoFisher Scientific, Waltham, MA, USA) at concentrations of 100 nM and 50 nM, respectively. Mimic and inhibitor were titrated to identify the optimal concentration of each effect. After transfection, cells were rested for 24 h before infection.

### 2.6. RNA Extraction

Cell samples were lysed for the determination of cytokine transcripts and viral copies. For the expression of cytokines, the RNA was obtained with an RNeasy-plus kit (Qiagen, Germantown, MD, USA), according to the manufacturer’s recommendations. For the expression of miRNAs, total RNA was obtained using RNAzol^®^ RT (Sigma-Aldrich, St. Louis, MO, USA) according to the manufacturer’s protocol.

### 2.7. Quantitative Real-Time Reverse Transcription-PCR (qRT-PCR)

miRNA was quantified from total RNA. Each sample was used to make cDNA using the Mir-X TM miRNA Fists-Strand Synthesis Kit (TaKaRa Biotechnology, San Jose, CA, USA). RT-qPCR was performed in duplicate using 10 nM of each MystiCq microRNA qPCR Assay Primer and the TB Green Advantage qPCR Premix (TaKaRa Biotechnology, San Jose, CA, USA). RT-qPCRs were run on a Quant Studio 12k PCR system following the manufacturer’s suggested cycling parameters (Applied Biosystems, Foster City, CA, USA). The comparative cycle threshold (ΔΔCT) was used to quantitate the expression of target miRNAs and normalized to the endogenous reference expression levels of transcripts RNU6-1 from corresponding uninfected cells or infected cells.

The interferon and proinflammatory cytokine expression was quantified: the first-strand cDNA was synthesized from total RNA using the LunaScript RT SuperMix Kit (New England Biolabs, Ipswich, MA, USA) according to the manufacturer’s instructions. cDNA fragments of interest were amplified using the PowerTrack SYBR Green Master Mix (ThermoFisher Scientific, Waltham, MA, USA). Primers (Integrated DNA Technologies) for interferon (IFN)-β1, IFN-λ2/3, IFN-α2, TNF-α, IL-6, IFIT1, IFIT2, IFIT3, MX1, and GAPDH were run on the QuantStudio™ 12k PCR system (Applied Biosystems, Foster City, CA, USA). The CT method (ΔΔCT) was used to quantitate the expression of target genes, normalized to the endogenous reference (GAPDH) expression levels of transcripts from corresponding uninfected or infected cells. The specificity of the reaction was determined by melting curve analysis of the amplification products.

The absolute HMPV viral quantification (viral copies/μL) was assessed by generating a standard curve prepared with a plasmid containing the HMPV N protein gene. Data were analyzed using QuantStudio™ 12k Flex Software Version 1.3.

### 2.8. Statistical Analysis

Statistical analyses were calculated by unpaired Student t-test and one-way analysis of variance (ANOVA) to ascertain the differences between the tested conditions, followed by post-tests to correct for multiple comparisons using GraphPad Prism 9.5.0 (GraphPad Software, Boston, MA, USA).

## 3. Results

### 3.1. Suseptibility of Macrophages to HMPV Infection

**Figure 1:**
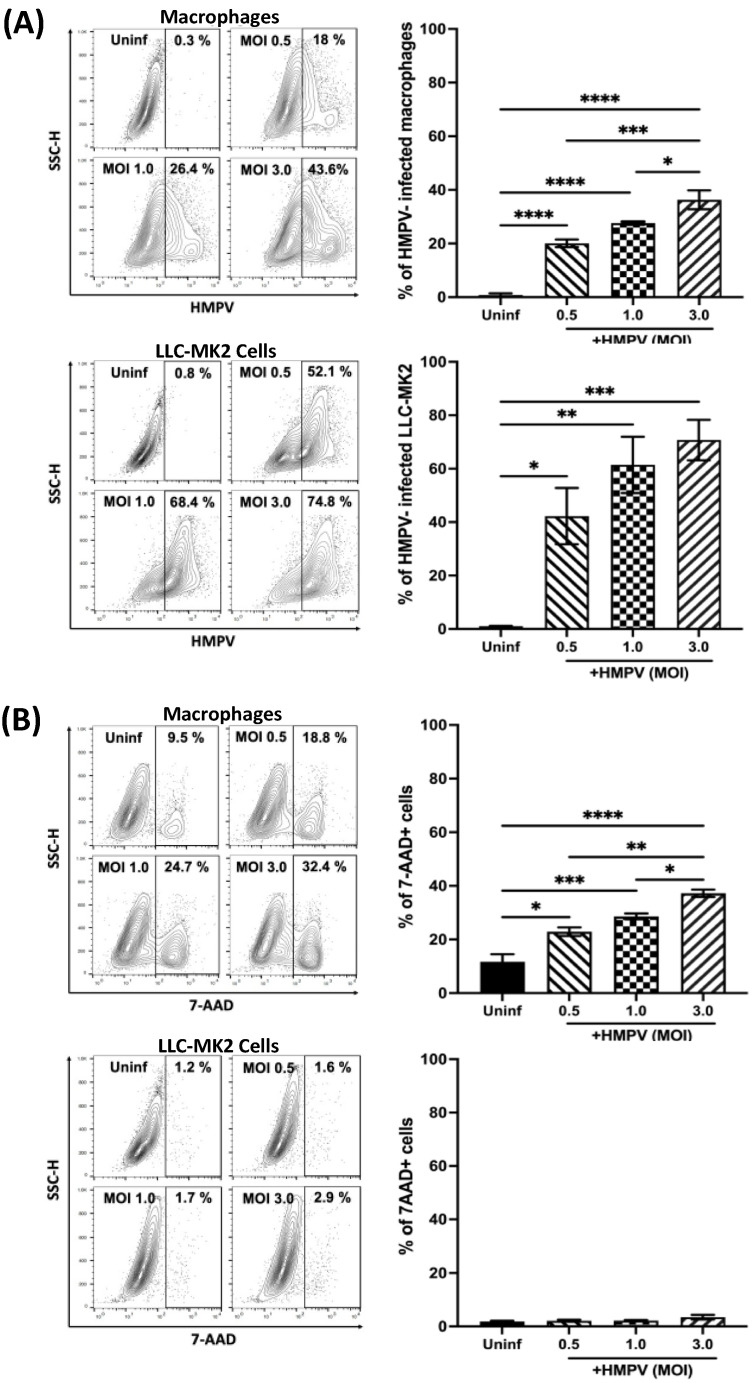
Susceptibility of macrophages to HMPV infection. THP-1-derived macrophages and LLC-MK2 cells were infected with HMPV at different MOIs. (**A**) The percentage of infected cells was determined by the expression of HMPV P protein. (**B**) Cell death was determined by 7-AAD staining. All samples were analyzed by flow cytometry and Flow Jo V10.9. Representative data of contour plot analyses are included. Graph bars represent the mean of 3 independent experiments ± SEM. Statistical differences were calculated using ANOVA followed by a post hoc Tukey’s multiple comparison test. * *p* < 0.05, ** *p* < 0.01, *** *p* ≤ 0.001, **** *p* ≤ 0.0001.

The susceptibility of macrophages to HMPV infection was analyzed in THP-1-derived macrophages. Cells were infected with HMPV at different MOIs for 24 h. After that time, the percentage of HMPV-infected macrophages was analyzed by viral antigen staining and flow cytometry. For comparison and validation of the infection data obtained in the macrophages, we also included the analysis of LLC-MK2 cells, a known permissive cell line for HMPV infection and the cell line used to propagate the virus [28]. As shown in Figure 1A (bar graph, upper panel), the percentage of infected macrophages increased in a dose-dependent manner from 20.0 ± 1.4% at an MOI of 0.5 to 27.6 ± 0.5% at an MOI of 1.0 to a maximum of 36.3 ± 3.5% at an MOI of 3.0. On the other hand, when compared to LLC-MK2, the percentages of infected cells were higher, observing 42.2 ± 10.5% (MOI of 0.5) and 61.4 ± 10.5% (MOI 1.0) to 70.7 ± 7.5% at an MOI of 3.0 (Figure 1A, bar graph, lower panel). Representative data analyses from both cell types are shown in the quantile contour plots (Figure 1A).

To define the optimal conditions for infecting macrophages with HMPV that could provide the maximum infectivity with the least cell damage, the percentage of dead cells after HMPV infection was determined in the same culture conditions as in Figure 1A. Cell death was assessed by 7-AAD staining followed by flow cytometry analysis. As shown in the bar graphs of Figure 1B, uninfected macrophages showed a percentage of 7-AAD positive cells of 11.4 ± 2.9%, while macrophages infected at an MOI of 3.0 exhibited the highest level of cell death (36.2 ± 2.1%). Cells infected at MOIs of 5.0 and 10.0 induced 42% and 43.4% cell mortality, respectively [29]. However, no significant difference between macrophages infected at an MOI of 0.5 (22.5 ± 1.9%) or 1.0 (27.8 ± 1.2%) was observed. On the other hand, the cell viability was minimally compromised in uninfected LLC-MK2 cells (1.6 ± 0.2%), which was marginally increased after HMPV infection at any of the tested MOIs to about 3%. The quantile contour plots show representative data analyses from each cell type. Overall, the combined viral infection and cell death experiments allowed us to determine that the best condition to infect macrophages with HMPV used the MOI of 1.0, which resulted in significant levels of HMPV-infected cells without the maximum cell mortality.

**Figure 2:**
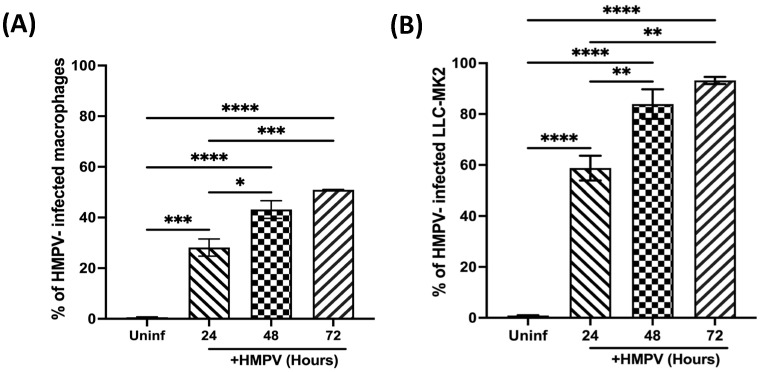
Susceptibility of macrophages to HMPV infection at different time points. (**A**) THP-1-derived macrophages were infected with HMPV at MOI 1.0. (**B**) LLC-MK2 cells were included as permissive reference cells. The percentage of infected cells was determined by the expression of HMPV P protein at different time points. All samples were analyzed by flow cytometry and Flow Jo V10.9. Graph bars represent the mean of 3 independent experiments ± SEM. Statistical differences were calculated using ANOVA followed by a post hoc Tukey´s multiple comparison test. * *p* < 0.05, ** *p* < 0.01, *** *p* ≤ 0.001, **** *p* ≤ 0.0001.

To further characterize the infection in macrophages, we assessed the influence of time on the susceptibility of macrophages to HMPV infection compared to LLC-MK2 cells (HMPV-susceptible cells). Macrophages were infected at an MOI of 1.0, and cells were stained 24, 48, and 72 h after infection, as mentioned above. Data shown in Figure 2A indicate that, at 24 h postinfection, 28.1 ± 3.4% of macrophages were infected with HMPV. That percentage was increased to 43.2 ± 3.5% at 48 h and 50.9 ± 0.1% at 72 h. For LLC-MK2 susceptibility to HMPV (Figure 2B), we observed that 58.7 ± 4.8% of cells were infected at 24 h, reaching a maximum of 93.2 ± 1.4% at 72 h. These findings demonstrate that macrophages are susceptible to HMPV infection but to a lesser degree than known permissive cells like LLC-MK2 cells.

### 3.2. miRNA Expression in HMPV-Infected Macrophages

**Figure 3:**
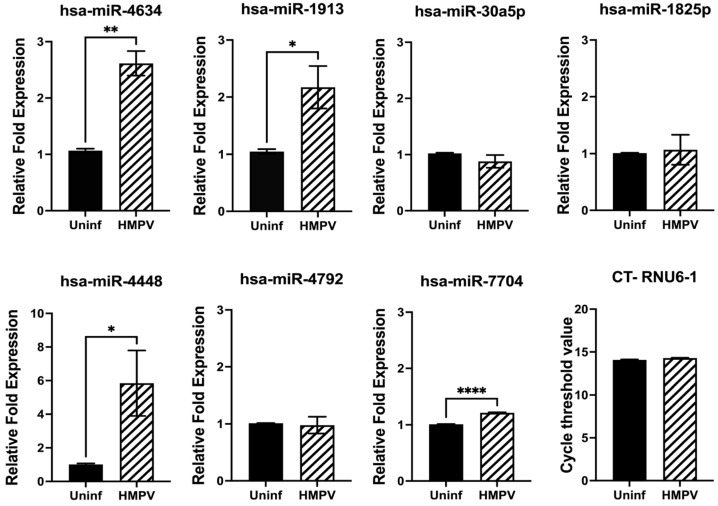
miRNA expression in macrophages infected with HMPV. Macrophages were infected with HMPV at an MOI of 1 for 24 h. miRNA expression was assessed by miRNA RT-qPCR and normalized to RNU6-1. Bar graphs represent mean of 3 independent experiments ± SEM. Statistical differences between infected and uninfected cells were calculated using unpaired Student *t*-test * *p* < 0.05, ** *p* < 0.01, **** *p* ≤ 0.0001.

Next, we investigated the expression of miRNAs in macrophages. THP-1-derived macrophages were infected with HMPV at an MOI of 1.0 for 24 h. The expression of miRNAs was assessed by RT-qPCR. Our results, shown in Figure 3, demonstrated that the expression of hsa-miR-4634 was significantly increased by HMPV, with a 2.6-fold induction over the uninfected cells (*p* < 0.01). Similarly, hsa-miR-1913 was also induced by HMPV (*p* < 0.05) but in a lower proportion than hsa-miR-4634 (2.1-fold). The expression of hsa-miR-7704 (1.2-fold) was also significant compared to uninfected cells, but its induction by HMPV infection was rather marginal. Finally, HMPV infection also induced the expression of hsa-miR-4448 by a 5.8-fold increase. However, the experiments had greater variability. Therefore, based on the expression level and experimental reproducibility, we focused this study on the analysis of hsa-miR-4634.

### 3.3. Functional Analysis of hsa-miR-4634 in Macrophages Infected with HMPV

**Figure 4:**
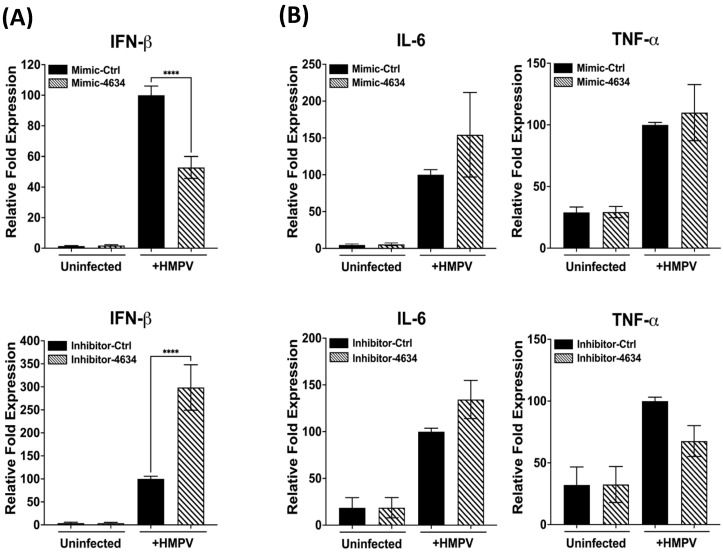
Effect of hsa-miR-4634 on interferon and proinflammatory cytokines expression. THP-1-derived macrophages were transfected by electroporation with hsa-miR-4634 mimic (100 nM) or inhibitor (50 nM). After 24 h of culture, macrophages were infected with HMPV at an MOI of 1 for 24 h. Total RNA was extracted to assess gene expression using RT-qPCR. (**A**) IFN-β expression of macrophages transfected with mimic hsa-miR-4634 (upper panel) or inhibitor hsa-miR-4634 (lower panel). (**B**) Expression of inflammatory cytokines in macrophages transfected with mimic or inhibitor hsa-miR-4634. Bar graphs represent the mean of three independent experiments ± SEM. Statistical differences between infected and uninfected cells were calculated using ANOVA, followed by Sidak’s multiple comparison test. **** *p* < 0.0001.

Based on the predominant expression of hsa-miR-4634 by HMPV (Figure 3), the function of this miRNA on the macrophage immunity in response to HMPV infection was investigated by using a mimic or inhibitor for hsa-miR-4634. The miR-4634 inhibitor (herein referred to as inhibitor-4634) is a single-stranded RNA molecule specific to inhibiting the function of hsa-miR-4634 by hybridizing with endogenous and mature hsa-miR-4634. At the same time, the miR-4634 mimic is a chemically modified RNA duplex that mimics the function of the endogenous hsa-miR-4634 (herein referred to as mimic-4634). The corresponding negative controls for either mimic-4634 or inhibitor-4634 are inert and do not modulate any genes after transfection into the cells. THP-1-derived macrophages were transfected with either a mimic or an inhibitor. After 24 h, cells were infected with HMPV at an MOI of 1.0 for 24 h. The expression of IFN-α2, IFN-β, and proinflammatory cytokines (IL-6 and TNF-α) was assessed by RT-qPCR. The data shown in Figure 4A (upper panel) indicate that adding hsa-miR-4634 (mimic) decreased HMPV-induced IFN expression. That effect was reversed in the presence of hsa-miR-4634 inhibitor (lower panel), where an increase in IFN-β expression was observed in the presence of hsa-miR-4634 inhibitor. The expression of IFN-α2 by HMPV infection in macrophages was not induced [30]. Furthermore, when assessing the effect of hsa-miR-4634 on the expression of proinflammatory cytokines IL-6 and TNF-α, no significant changes caused by the hsa-miR-4634 mimic or inhibitor were observed, as shown in Figure 4B. Overall, these data suggest that hsa-miR-4634 regulates type I IFN response in macrophages.

### 3.4. Interferon-Stimulated Gene (ISG) Expression in Macrophages Treated with Mimic and Inhibitor of hsa-miR-4634

**Figure 5:**
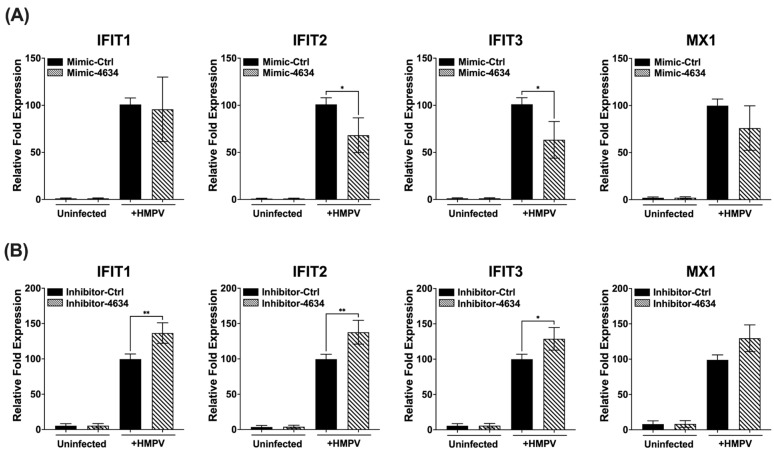
Effect of hsa-miR-4634 on interferon-stimulated genes in macrophages. THP-1-derived macrophages were transfected by electroporation with (**A**) hsa-miR-4634 mimic (100 nM) or (**B**) inhibitor (50 nM) (see Methods). After 24 h of culture, macrophages were infected with HMPV at an MOI of 1.0 for 24 h. Total RNA was extracted to assess gene expression of IFIT1, IFIT2, IFIT3, and MX1 by using RT-qPCR. Graph bars represent the mean of four independent experiments ± SEM. Statistical differences between infected and uninfected cells were calculated using ANOVA, followed by Sidak’s multiple comparison test. * *p* < 0.05; ** *p* < 0.01.

Interferon-stimulated genes (ISGs) are induced after the activation by type I or III interferons, IFNAR1/IFNAR2 and IFNLR1/IL-10R2, respectively [31]. The effect of hsa-miR-4634 on ISGs IFIT1, IFIT2, IFIT3, and MX1 was investigated after previous demonstrations showed this miRNA changed the gene expression of IFNs in macrophages. Our results demonstrated that hsa-miR-4634 affected mainly the expression of IFIT2 and IFIT3, where it was observed that the addition of mimic-4634 significantly decreased the expression of IFIT2 and IFIT3. The MX1 expression modified by the mimic was not significantly reduced (Figure 5A). On the other hand, the presence of inhibitor-4634 induced an increased expression of IFIT1, IFIT2, and IFIT3. The increase observed in MX1 expression by the effect of the inhibitor was not significant (Figure 5B). The expression of IFIT1 was altered by the transfection of inhibitor-4634, but no effect was observed with mimic-4634. These findings suggest that hsa-miR-4634 regulates the expression of IFIT2 and IFIT3 in macrophages.

### 3.5. Functional Analysis of hsa-miR-4634 in Epithelial Cells Infected with HMPV

**Figure 6:**
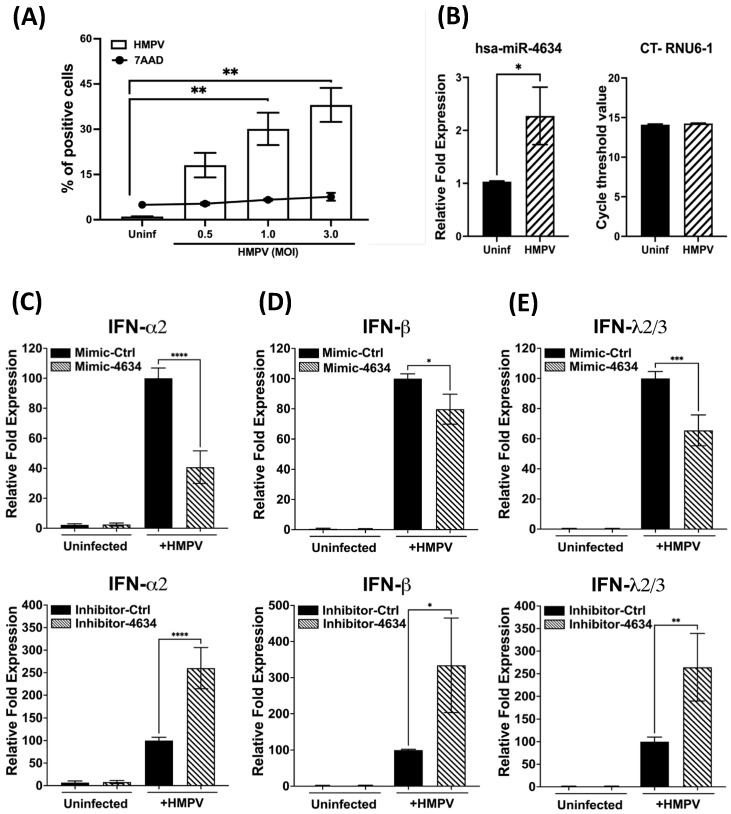
Effect of hsa-miR-4634 on interferon expression in epithelial cells. (**A**) A549 cells were infected at MOI 0.5, 1.0, and 3.0, and the percentage of HMPV-positive cells and cell death was quantified by flow cytometry. (**B**) A549 cells were infected with HMPV at an MOI of 3.0 for 24 h, and the expression of hsa-miR-4634 was assessed by RT-qPCR. (**C**–**E**) A549 cells were transfected by electroporation with mimic-4634 (100 nM) or inhibitor-4634 (50 nM). After 24 h of culture, cells were infected with HMPV at an MOI of 3.0 for 24 h. Total RNA was extracted to assess the expression of IFN-α2, IFN-β, and IFN-λ2/3 by qPCR. Bar graphs represent mean of three independent experiments ± SEM. Statistical differences were calculated using the Student *t*-test (**B**) or ANOVA followed by Sidak’s multiple comparison test (**A**,**C**–**E**). * *p* < 0.05; ** *p* < 0.01, *** *p* < 0.001, **** *p* ≤ 0.0001.

Given the tropism of HMPV for epithelial cells [13,19,32,33], the regulatory effect of hsa-miR-4634 in A549 cells was investigated. First, the susceptibility of the cells to infection by HMPV was characterized. As shown in Figure 6A, it was observed that the infection occurs in a dose-dependent fashion. The percentage of infected cells at the MOI of 0.5 was 18.1% and increased to 30.13% at an MOI of 1.0, reaching 38.1% at the MOI of 3.0. Cell viability was maintained at a low percentage since the percentage of dead cells was not significantly increased from uninfected cells (4.9 ± 0.8%) to cells infected with HMPV at an MOI of 3.0 (7.6 ± 1.2%) (Figure 6A). Based on the combined infection and cell death results, the rest of the experiments with epithelial cells were conducted by infecting them at an MOI of 3.0, yielding the maximum percentage of infection. After assessing hsa-miR-4634 expression in epithelial cells, our data indicate that HMPV induced significant expression of hsa-miR-4634 in epithelial cells (Figure 6B). Next, A549 cells were transfected with a mimic or inhibitor for hsa-miR-4634 under similar experimental conditions as in Figure 4. The relative expression of IFN-α2, IFN-β, and IFN-λ2/3 was evaluated by RT-qPCR. Functional assay results reported in Figure 6C (IFN-α2), 6D (IFN-β), and 6E (IFN-λ2/3) indicate that when the effect of hsa-miRNA-4634 was blocked with inhibitor-4634, the expression of IFNs was significantly increased to more than twice its expression when compared with the cells treated with the inhibitor control. The opposite effect was observed when the cells were treated with mimic-4634, where the expression of IFNs was significantly reduced. These data confirm the regulatory effect of hsa-miR-4634 on the interferon response in epithelial cells, as observed above in macrophages (Figure 5).

### 3.6. hsa-miR-4634 Regulates the Viral Replication in Macrophages and Epithelial Cells

**Figure 7:**
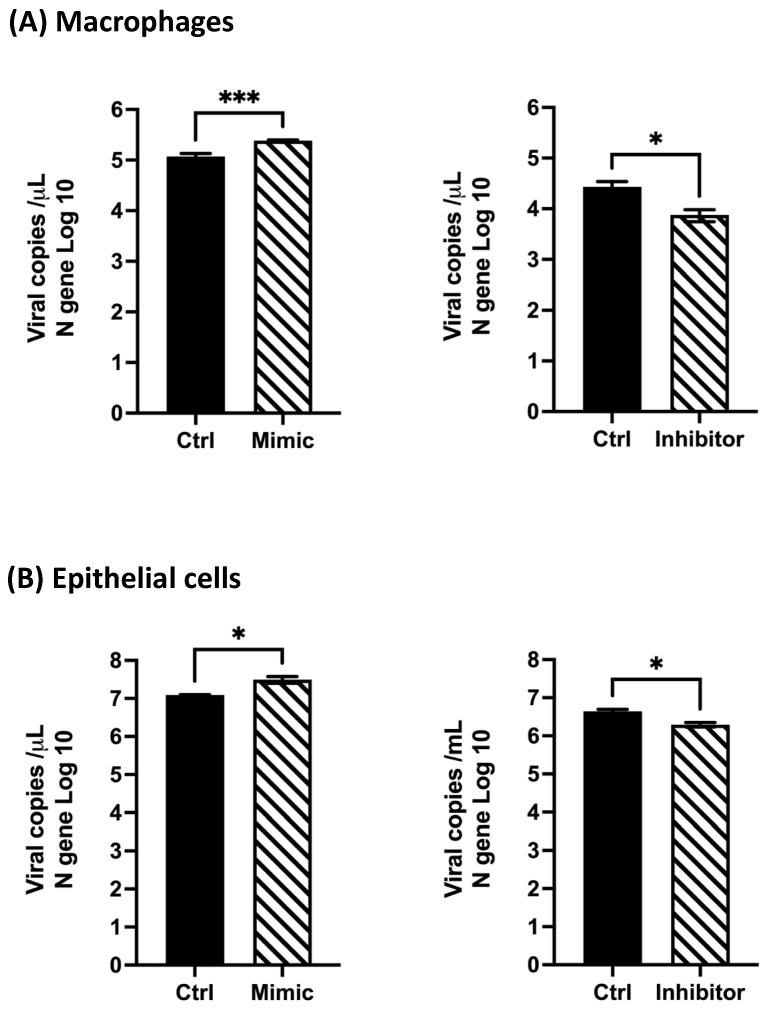
Effect of hsa-miR-4634 on viral replication in macrophages and epithelial cells. Viral copies of the HMPV N gene were quantified by RT-qPCR. Cells were transfected by electroporation with mimic (100 nM) and inhibitor (50 nM). (**A**) Macrophages and (**B**) epithelial cells were infected for 24 h with HMPV at MOI 1 or MOI 3, respectively. Bar graphs represent the mean of two independent experiments with similar results ± SEM. Statistical differences were calculated using the Student *t*-test. * *p* < 0.05, *** *p* <0.001.

Based on the observed effect of hsa-miR-4634 on the expression of ISGs (Figure 5), further analysis of the antiviral response against HMPV was further investigated. THP-1-derived macrophages and epithelial cells were transfected with mimic-4634 or inhibitor-4634 and infected with HMPV. Viral replication was quantified by qPCR to determine the absolute number of viral copies (HMPV N gene). Our findings showed that the mimic of hsa-miR-4634 increased viral replication by approximately 0.31 log10 in macrophages, while the inhibitor-4634 reduced the number of viral copies by about 0.55 log10 (Figure 7A). In epithelial cells, it was observed that mimic-4634 increased HMPV replication by ~0.4 log10, and the inhibitor decreased it by about 0.35 log10 (Figure 7B). These findings demonstrate that hsa-miR-4634 contributes to HMPV replication in macrophages and epithelial cells.

## 4. Discussion

Human metapneumovirus (HMPV) is a respiratory virus that causes clinical manifestations in the upper and lower respiratory tract and triggers several aspects of the host immune response [1]. Moreover, the virus has developed multiple mechanisms of evasion of the immune system, including the alteration of the interferon (IFN) response [34,35,36]. However, the effect of HMPV on the IFN response in infected cells warrants further investigation. Here, we demonstrate the contribution of hsa-miR-4634 to the viral evasion of innate immunity in macrophages.

Macrophages in the respiratory tract are considered key cells that participate in the defense against respiratory viruses through diverse mechanisms [16]. Upon infection, macrophages are activated to induce the expression of type I IFN and proinflammatory cytokines such as IL-6 and TNF-α [1,9]. Although macrophages are one of the first lines of defense in the respiratory tract [16], information on the interaction of these cells and HMPV is limited. Our data indicate that THP-1 monocyte-derived macrophages are susceptible to HMPV infection, with ~28% of infected cells at an MOI of 1.0 after 24 h (Figure 1A). These observations resemble those reported in human monocyte-derived macrophages (MDM) infected with HMPV NL/17/00, where a maximum of 23% of infected cells was observed [37]. On the other hand, A549 epithelial cells were more susceptible to HMPV infection, resulting in 38% infected cells. Comparable data were reported in A549 cells infected with HMPV NL/1/00 at an MOI of 2.0 for 24 h, where ~23% of cells were positive for HMPV in a flow cytometry analysis [38]. However, the susceptibility of A549 to HMPV might vary depending on the HMPV strain that is used, as reported in a study using 13 HMPV clinical isolates with variable levels of infectivity in A549 cells (~3–80%), although the percentage in A549 cells was calculated relative to VeroE6 cells as 100% [39].

Alveolar macrophages (AM) are reported to be the primary source of interferon production in response to RNA viruses [9,40]. In the case of HMPV infection, experiments in mice depleted of AM demonstrated that macrophages contribute to the IFN response [20]. However, the molecular mechanisms of IFN regulation in HMPV-infected macrophages are less known. In the present work, we examined the expression of miRNAs in macrophages. We found that HMPV induces significant expression of hsa-miR-4634, hsa-miR-1913, hsa-miR-7704, and hsa-miR-4448 with a predominant expression of hsa-miR-4634 (Figure 3). According to MirTarBase [41], 35 predicted target genes for hsa-miR-4634 have been validated by NGS and CLIP-Seq. Many of those targets are related to cancer. In fact, the expression of hsa-miR-4634 was linked to breast cancer [42] and reported as a tumor suppressor in lung cancer [43]. However, to the best of our knowledge, the current work is the first to report an effect of hsa-miR-4634 on the innate antiviral immune response. Nevertheless, additional in silico and experimental analyses will be needed to dissect the underlying molecular mechanism involved in the regulatory effect of hsa-miR-4634 on the IFN response observed in HMPV-infected cells.

The overexpression of hsa-miR-1913 was also associated with multiple forms of cancer [44,45,46,47,48,49], similar to hsa-miR-7704 [50,51,52]. However, the role of these miRNAs in HMPV infection warrants additional research.

Based on the strongest validated target for hsa-miR-4448 reported in miRTarBase, hsa-miR-4448 targets CCDC88A, a gene that codes for GIV or Girdin. GIV is a multimodular scaffold protein that modulates G-protein activity [53] that has been associated with the regulation of the Akt/mTOR signaling pathway [54] and is highly expressed in macrophages [55]. The presence of GIV in murine macrophages was reported to inhibit the expression of proinflammatory cytokines IL-6 and IL-1β during bacterial infection [55]. However, the effect of HMPV infection on GIV expression in infected macrophages remains to be investigated.

Further functional assays using the mimic or inhibitor revealed that hsa-miR-4634 inhibits the IFN response and ISG expression, influencing viral replication in macrophages, suggesting a regulatory mechanism by which the virus may evade the host antiviral immune response. In this regard, hsa-miR-4634 may represent a desirable target for future therapeutic applications to modulate the IFN response. A similar effect was observed in the hepatitis C virus (HCV), a single-stranded positive-sense virus that is also capable of regulating the IFN response. HCV can induce host microRNAs miR-208b and miR-499a-5p, myomiRs specifically expressed by HCV that suppress both type I and type III IFN responses [56]. However, to the best of our knowledge, this is the first report to demonstrate that HMPV dampens the IFN response in macrophages through the expression of host miRNAs. Nonetheless, the present work is limited to the functional assays of hsa-miR-4634, and future research is warranted to explore the functional role of miR1913, 7704, and 4448 in HMPV infection.

The secretion of proinflammatory cytokines during HMPV has been associated with disease severity [15,57]. Therefore, we investigated the effect of hsa-miR-4634 in TNF and IL-6 expression. However, unlike to the IFN response, we did not find a regulatory effect of hsa-miR-4634 on the expression of the inflammatory cytokines. These observations might suggest that hsa-miR-4634 regulates the IRF signaling pathway but not the NF-kB pathway. However, future research is needed to demonstrate such a possibility.

Epithelial cells are known to produce type I and type III IFNs, which activate an antiviral state of the infected cell, preventing viral replication in an autocrine and paracrine manner. Therefore, we evaluated the effect of hsa-miR-4634 on the modulation of IFN-α2, β, and λ2/3. Our findings indicate that hsa-miR-4634 also inhibits the IFN response in epithelial cells and increases HMPV replication. The regulatory effect of hsa-miR-4634 on the IFN response in epithelial cells has not been reported in other miRNAs induced by HMPV. However, using a similar model of HMPV infection, Deng et al. demonstrated that miR-let-7f inhibits HMPV replication, suggesting that miR-let-7f may have an antiviral effect on the infected cells [58]. Overall, these data highlight the importance of functional characterization of miRNAs in HMPV infection.

Furthermore, beyond the antiviral response, IFNs are regarded as necessary for other immunoregulatory functions, shaping innate and adaptive immunity as well as maintaining tissue homeostasis and integrity [59,60,61,62,63], making it crucial to understand the impact of viral infections on the expression of these molecules since an altered IFN response could have an impact on the overall host immune response.

In summary, the current findings provide new insights into the complex mechanisms governing the regulation of IFN responses in HMPV infection and highlight the relevance of miRNAs in the host immune response to respiratory viruses, which is essential for furthering our understanding of the complexity of miRNAs and their therapeutic potential for HMPV and possibly other viral infections.

## Data Availability

The data supporting the conclusions of this research manuscript are all present within the article.
